# Squalene Epoxidase: Its Regulations and Links with Cancers

**DOI:** 10.3390/ijms25073874

**Published:** 2024-03-30

**Authors:** Lin Zhang, Zheng Cao, Yuheng Hong, Haihua He, Leifeng Chen, Zhentao Yu, Yibo Gao

**Affiliations:** 1 Department of Thoracic Surgery, National Cancer Center/National Clinical Research Center for Cancer/Cancer Hospital & Shenzhen Hospital, Chinese Academy of Medical Sciences and Peking Union Medical College, Shenzhen 518116, China; 2 Department of Pathology, National Cancer Center/National Clinical Research Center for Cancer/Cancer Hospital, Chinese Academy of Medical Sciences and Peking Union Medical College, Beijing 100021, China; 3 Department of Thoracic Surgery, National Cancer Center/National Clinical Research Center for Cancer/Cancer Hospital, Chinese Academy of Medical Sciences and Peking Union Medical College, Beijing 100021, China; 4 Department of Oncology, Renmin Hospital of Wuhan University, Wuhan 430060, China; 5 Central Laboratory & Shenzhen Key Laboratory of Epigenetics and Precision Medicine for Cancers, National Cancer Center/National Clinical Research Center for Cancer/Cancer Hospital & Shenzhen Hospital, Chinese Academy of Medical Sciences and Peking Union Medical College, Shenzhen 518116, China; 6 State Key Laboratory of Molecular Oncology, National Cancer Center, National Clinical Research Center for Cancer, Cancer Hospital, Chinese Academy of Medical Sciences and Peking Union Medical College, Beijing 100021, China; 7 Laboratory of Translational Medicine, National Cancer Center/National Clinical Research Center for Cancer/Cancer Hospital, Chinese Academy of Medical Sciences and Peking Union Medical College, Beijing 100021, China

**Keywords:** SQLE, mevalonate pathway, cholesterol metabolism, ferroptosis, cancer

## Abstract

Squalene epoxidase (SQLE) is a key enzyme in the mevalonate–cholesterol pathway that plays a critical role in cellular physiological processes. It converts squalene to 2,3-epoxysqualene and catalyzes the first oxygenation step in the pathway. Recently, intensive efforts have been made to extend the current knowledge of SQLE in cancers through functional and mechanistic studies. However, the underlying mechanisms and the role of SQLE in cancers have not been fully elucidated yet. In this review, we retrospected current knowledge of SQLE as a rate-limiting enzyme in the mevalonate–cholesterol pathway, while shedding light on its potential as a diagnostic and prognostic marker, and revealed its therapeutic values in cancers. We showed that SQLE is regulated at different levels and is involved in the crosstalk with iron-dependent cell death. Particularly, we systemically reviewed the research findings on the role of SQLE in different cancers. Finally, we discussed the therapeutic implications of SQLE inhibitors and summarized their potential clinical values. Overall, this review discussed the multifaceted mechanisms that involve SQLE to present a vivid panorama of SQLE in cancers.

## 1. Background

Cholesterol is a major lipid constituent of biological membranes and plays a critical role in cellular processes such as intracellular transport, cell signaling, adhesion, membrane fluidity, and permeability [1]. Cholesterol also acts as a precursor of bile acid, and its oxidative effect allows for the biosynthesis of steroid hormones in steroid-producing tissues [2]. The aforementioned characteristics make cholesterol crucial for the growth and survival of mammalian cells. Accumulation of cholesterol in malignant tumors is a well-known phenomenon; cholesterol and low-density lipoprotein expression have been considered as risk factors, as they are reported as drivers of tumor growth and are associated with worse prognoses in breast, prostate, brain, and colorectal cancers [3]. Mammalian cells have two main ways of obtaining cholesterol: exogenous uptake and endogenous synthesis [2]. A variety of daily foods, such as eggs, animal offal, and seafood, contain cholesterol; the cholesterol uptake pathway consists of NPC1L1 protein-mediated absorption from the food, which enters the intestinal lumen, as well as LDLR-mediated subsequent absorption from the blood [1]. In contrast, tumor cells require excess cholesterol and intermediates of the cholesterol biosynthesis pathway to maintain their proliferation; therefore, abnormalities in cholesterol biosynthesis are strongly associated with tumorigenesis [2].

Cholesterol is a ubiquitous sterol, present in vertebrates, with multiple biological functions, and the cholesterol synthesis pathway has been characterized as a carefully controlled pathway that starts with acetyl coenzyme A (acetyl-CoA) and involves over 20 enzymes [4]. Steps in this process are tightly regulated and some intermediates produced can be transferred and used as precursors for the biosynthesis of other bioactive compounds [5]. There are two rate-limiting enzymes in the biosynthesis pathway of cholesterol: 3-hydroxy-3-methylglutaryl coenzyme A reductase (HMGCR) and squalene epoxidase (SQLE) [5]. The step for reduction in HMG-CoA by HMGCR is most critical and the important roles that HMGCR plays in physiological conditions and tumors have been thoroughly investigated [1,2]. Its most famous inhibitor, statins, are the most widely used cholesterol-lowering drug. Despite the many observational and preclinical studies that have revealed a growing number of pathways and cancer therapy targets, there is a consistent lack of solid data from prospective, randomized trials. Atorvastatin exhibited one of the few oncological benefits in patients with head and neck cancers or protective effects on healthy tissues exposed to chemo-/radiotherapy [6,7]. However, the inhibition of HMGCR could also reduce non-steroidal products such as coenzyme Q which is necessary for T cells to make metabolic adaptations and enhance anti-tumor immunity [8]. This prompted us to wonder whether the inhibition of enzymes downstream HMGCR, such as SQLE, was able to generate such survival benefits and avoid these disadvantages.

SQLE is the second key enzyme in cholesterol biosynthesis [9]. However, compared to HMGCR, SQLE has gained much less attention. SQLE catalyzes the first oxygenation step of cholesterol biosynthesis, the conversion of squalene to 2,3-oxidosqualene, a metabolite that is subsequently cyclized to form lanosterol or cycloartenol [10]. This reaction lies immediately after the first committed step of cholesterol synthesis, i.e., the formation of squalene by squalene synthase, and directly precedes the cyclization step that forms the first sterol intermediate, lanosterol [10]. In recent years, more and more studies have shown that SQLE is elevated in cancers and the dysregulation of SQLE could result in cholesterol metabolism disorder, which constitutes a key dysregulated event in cancers [11,12]. In addition, the turnover of SQLE has been linked to ferroptosis, which shed a light on novel therapeutic implications in cancers [13,14]. In the current review, we illustrate the regulations of SQLE and its roles in cancer development and progression and introduce the current understanding of its inhibitors, aiming to provide novel insights in developing targets for cancer therapy.

## 2. The Structure and Topology of SQLE

**Figure 1:**
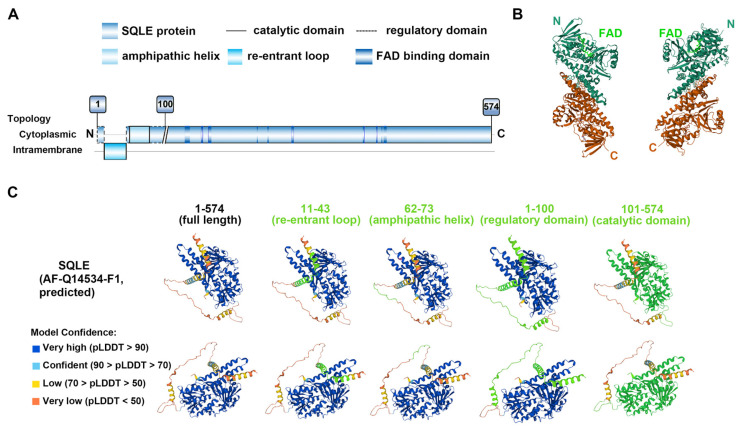
**Structure of SQLE**. (**A**) The overall structure and topology of human SQLE. The first 100 amino acids of SQLE (N100) constitute the regulatory domain, while the remaining 474 amino acids make up the catalytic domain. The amphipathic helix in N100 attaches reversibly to the endoplasmic reticulum membrane depending on cholesterol levels. The substrate-binding domains exist in the catalytic domain, and FAD-binding domains intersperse within the primary structure of the catalytic part. With reference to the study by Chua NK et al. [9,18], Brown AJ et al. [21], and Padyana AK et al. [19]. (**B**) Human SQLE structure with FAD, from the PDB database [22,23]. PDB number 6C6R (PDB https://doi.org/10.2210/pdb6C6R/pdb (accessed on 14 September 2023)). Amino acids 118–574 of SQLE are shown. The study was conducted by Padyana AK et al. [19]. (**C**) Predicted Human SQLE structure from the UniProt database [24]. Protein identifier AF-Q14534-F1. Full length of SQLE and different domains on the protein, displayed from different perspectives; the domains are highlighted in bright green.

SQLE is a 574-amino acid protein weighing 64 kDa and is encoded by the *SQLE* gene, which is located at chromosome 8q24.1 (chr8q24.1) and spans approximately 23.8 kilobase pairs [9,15]. The gene is organized into 11 exons with 10 introns [16]. The hydrophobic nature of SQLE makes it difficult for a crystal structure to be obtained, so a biochemical approach was taken to elucidate its membrane topology [17]. SQLE protein inserts into the endoplasmic reticulum (ER) membrane and has a sinuous topology. The first 100 amino acids on the N-terminus (N100) constitute the regulatory domain, while amino acids 101–574 compose the catalytic domain (Figure 1A,C). The N100 regulatory domain represents the region responsible for the end product-mediated degradation of SQLE [18]. The structure of the catalytic domain of human SQLE was unveiled by Padyana and colleagues: they confirmed the crystal structures of the FAD-bound human SQLE (Figure 1B) and identified two potent inhibitors, NB-598 and Cmpd-4 [19].

An evident characterization of SQLE protein is the re-entrant loop in the first 100 amino acids of the N-terminus, which is embedded in the ER membrane (Figure 1A) [17]. Both the N- and C-termini of N100 are cytosolic, and the re-entrant loop spans from 24 to 33 residues [18]. It is believed that the insertion of SQLE in the ER membrane was implemented post-transcriptionally because the cholesterol-dependent degradation of SQLE was mediated by E3 ubiquitin ligase and proteasome, and the truncated N100 was enough for its degradation [20]. Other than that, an amphipathic helix also locates in this region and is responsible for its ER membrane anchoring [9]. The helix extends from residues 62 to 73 and attaches reversibly to the ER membrane depending on cholesterol levels; when cholesterol becomes excessive, the helix is ejected and unravels to expose a hydrophobic patch that serves as a degradation signal [18]. In addition to N100, the N-terminal-truncated protein contains three distinct domains, the FAD-binding domain, the substrate-binding domain, and a C-terminal helical membrane-binding domain [19]. The binding of the substrate squalene or inhibitor NB-598 occurs in the substrate/inhibitor-binding domain in amino acids 100–517 [9].

## 3. The Role of SQLE in Cholesterol Biosynthesis

**Figure 2:**
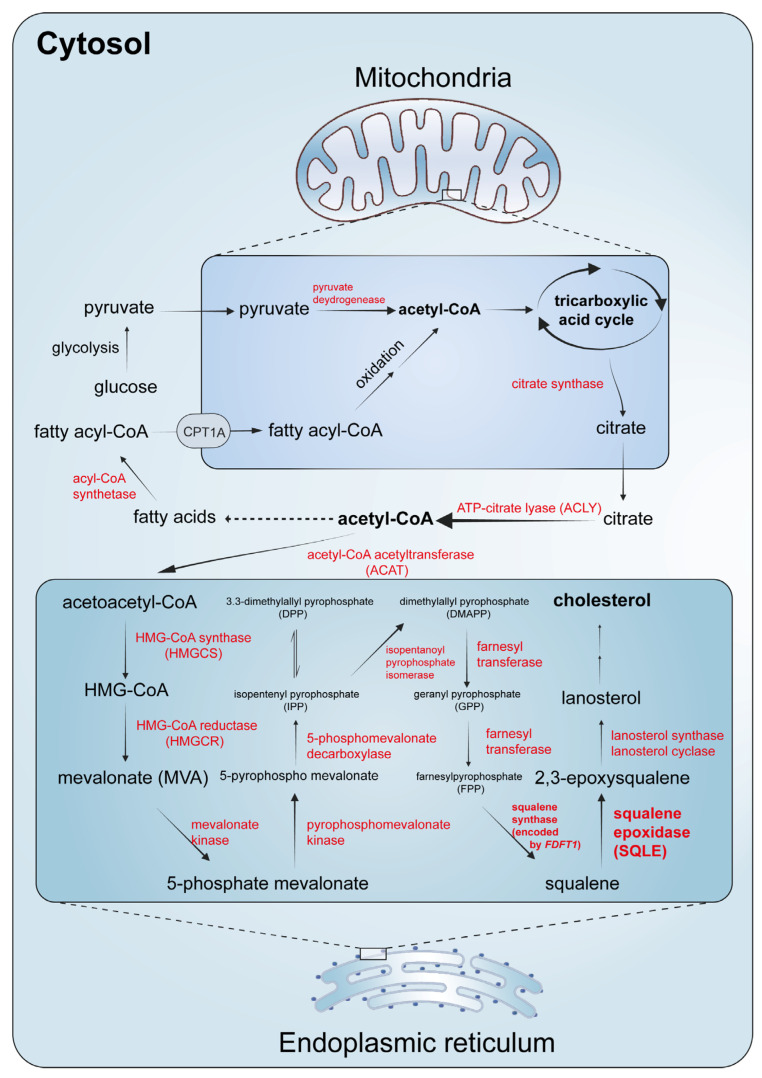
Biosynthesis of cholesterol. With reference to the studies by Chua NK et al. [9] and Gobel A et al. [3].

Cholesterol is a type of lipid that constitutes an essential component of mammalian cell membranes and plays a crucial role in maintaining normal cell function [25]. There are two main sources of cholesterol in our body: one is through dietary intake, known as exogenous cholesterol or dietary cholesterol, and the other is through de novo biosynthesis, known as endogenous cholesterol [1]. The process of cholesterol biosynthesis is regulated by several crucial factors, including HMGCR, SQLE, and sterol regulatory element-binding protein (SREBP) [25]. The biosynthesis starts from acetyl-CoA, a metabolic intermediate that supports the tricarboxylic acid cycle, with the involvement of nearly 30 enzymatic reactions (Figure 2) [1]. Pyruvate produced by glycolysis in the cytoplasm and fatty acid oxidation in the mitochondria are two important sources of acetyl-CoA. Acetyl-CoA is unable to cross the mitochondrial membrane and is synthesized into citrate by citrate synthase to be exported from the mitochondria [26]. Citrate is then converted to acetyl-CoA by ATP citrate lyase, providing the fundamental two-carbon building block for both fatty acid synthesis and cholesterol synthesis [26]. Cholesterol biosynthesis begins, and two molecules of acetyl-CoA are composed into acetoacetyl-CoA by acetyl-CoA acetyltransferase. Subsequently, a third acetyl-CoA molecule is synthesized into HMG-CoA by HMG-CoA synthase. In the next step, HMGCR is involved to produce mevalonate and constitutes one of the rate-limiting steps in cholesterol synthesis [25,27].

Mevalonate then undergoes phosphorylation by mevalonate kinase and phosphomevalonate kinase and is subsequently metabolized to 5-pyrophosphomevalonate. Following that, isopentenyl pyrophosphate (IPP) and its isomer 3,3-dimethylallyl pyrophosphate are formed by 5-phosphomevalonate decarboxylase; IPP is converted to dimethylallyl pyrophosphate (DMAPP) by isopentanoyl pyrophosphate isomerase, and DMAPP is used together with IPP as the materials for condensation into the fifteen-carbon farnesyl pyrophosphate (FPP) [1,3]. FPP serves as the basic product for squalene production and squalene is formed by the fusion of two FPP molecules; the reaction is catalyzed by squalene synthase [28]. Squalene is then converted to 2,3-epoxysqualene by SQLE, which constitutes the first oxygenation step in cholesterol synthesis [15]. Lanosterol synthase and lanosterol cyclase are subsequently involved and transform 2,3-epoxysqualene into lanosterol, an intermediate product that can be converted into cholesterol in more than twenty steps totally (Figure 2) [1]. The whole process is regulated by a negative feedback mechanism with the downstream products [1].

## 4. The Regulation of SQLE

### 4.1. Regulation by Cholesterol

Cholesterol biosynthesis is a tightly regulated process [5], and so is SQLE expression (Table 1). The regulation of SQLE occurs at several different levels. Primarily, the expression of SQLE can be controlled by its end product, cholesterol [20]. The interaction between cholesterol and SQLE was confirmed in a study using a chemoproteomic strategy that involved clickable, photoreactive sterol probes in combination with quantitative mass spectrometry; the study globally mapped cholesterol–protein interactions directly in living cells and identified SQLE as one of the proteins [29]. This regulation by cholesterol is dependent on the SQLE N100 regulatory domain (Figure 1A), a cholesterol-responsive degron that is responsible for cholesterol-accelerated degradation [20]. Mechanistically, cholesterol induces subtle conformational changes in the cytosolic residues within the N100 re-entrant loop (Figure 1A), followed by the deformation of the amphipathic helix (residues Gln62-Leu73) with increased cholesterol in the ER membrane [18]. The amphipathic helix is indispensable for the cholesterol-mediated regulation of SQLE as it attaches reversibly to the ER membrane and serves as a degradation signal [18]. Since SQLE N100 lacks the obvious structure for membrane attachment in its second half, it is possible that the amphipathic helix is only superficially associated with the ER membrane, as enlightened by studies on other lipid-binding amphipathic helices [18,30,31]. It is likely that increased cholesterol levels in the membrane can thicken the membrane, due to cholesterol’s condensing effect [32] and induce the dissociation of the partially associated amphipathic helix, leading to proteasomal degradation of SQLE and interruption of cholesterol biosynthesis. If the attachment of SQLE to the membrane were stronger, increased cholesterol would be unlikely to lead to the dissociation of the helix from the membrane [18]. Furthermore, cholesterol maintains its own homeostasis not only via direct protein interactions, but also by altering membrane properties; this finding is supported by the compelling evidence that enantiomeric cholesterol, which exerts membrane effects but not specific interactions, also elicits SQLE homeostatic responses [33]. In addition to the end product, cholesterol, squalene is also involved in the regulation of SQLE [34]. Squalene, the direct substrate of SQLE, can directly bind to N100, reducing the interaction with and ubiquitination by MARCH6, and mediate the stabilization of SQLE at the ER membrane [34].

### 4.2. Transcriptional Regulation

**Figure 3:**
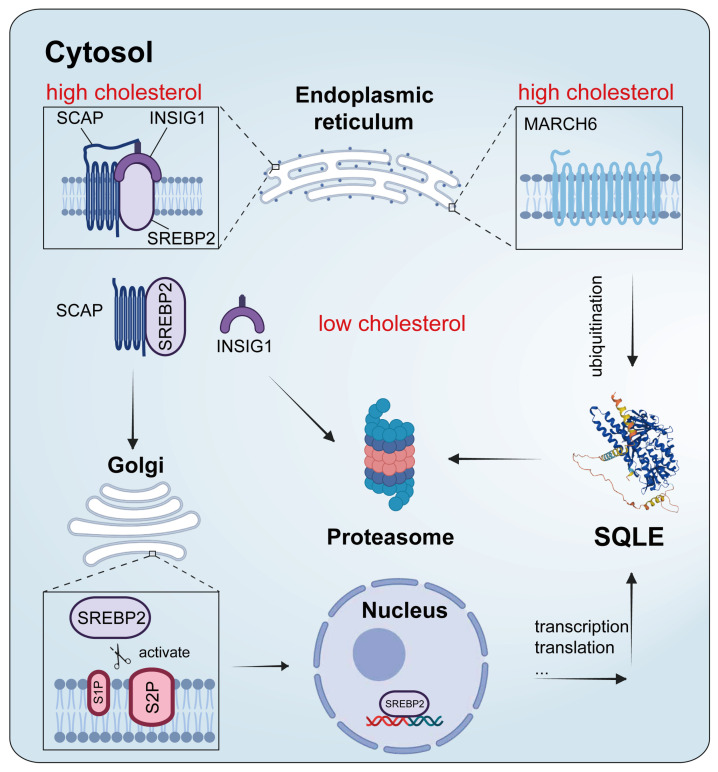
An overview of SQLE regulation.

The SREBP pathway serves as a master regulator in cholesterol de novo synthesis and functions at a transcriptional level [1,5,25]. The identification of SREBP was a breakthrough in understanding the regulation of cholesterol biosynthesis pathway genes [37]. SREBP transcription factors are synthesized as inactive precursors at the ER membrane and the N-terminal sequences of SREBP belong to the basic helix loop–helix–leucine zipper protein superfamily [1]. At the ER membrane, they are bound to SREBP cleavage-activating proteins (SCAPs), known as sterol sensors. SREBP2 is the major isoform involved in regulating cholesterol homeostasis and SQLE is a direct target of SREBP2 (Figure 3) [52]. When cholesterol levels in the ER exceed a critical threshold, SCAP undergoes a conformational change and binds to the tethering protein, INSIG1, which traps SREBP2 in the ER in its inactive precursor form [1]. When cholesterol levels are low, INSIG1 dissociates from SCAP and is degraded by the proteasome, which facilitates SCAP to escort SREBP2 to the Golgi apparatus, where the N-terminal of it can be proteolytically cleaved by proteases S1P and S2P; the cleaved SREBP2 then enters the nucleus to bind to the sterol regulatory element (SRE) sequence in the promoters of multiple target genes and induce the expression of them (Figure 3) [3,33,37]. So far, the SRE sequence has been found in many cholesterol biosynthesis genes such as squalene synthase, farnesyl diphosphate synthase, fatty acid synthase, acetyl coenzyme A carboxylase genes, and SQLE [16,53,54,55,56].

It has been revealed that SREBPs are weak transcriptional activators that need to cooperate with other regulators for robust induction, and the presence of other regulators may anchor SREBP to the DNA and make sustained interaction [57,58,59]. These regulators include NF-Y which regulates various cholesterol homeostasis genes including HMG-CoA synthase, HMGCR, and farnesyl diphosphate synthase [38,60], Sp1 which binds to region I (−276 bp/−176 bp) and region II (−86 bp/+25 bp) of SQLE [39], and YY1 which has been shown to bind to the proximal promoters of the genes encoding HMG-CoA synthase, FPP synthase, and the LDL receptor [61]. In a previous study, Nagai et al. showed that the sequence −207 to −192 base pairs of *SQLE* gene contained NF-Y binding sites [16]. In another study, to identify an SREBP2 responsiveness region, researchers used a pre-defined cell-based luciferase reporter assay that involved reporter constructs containing progressive deletions of the promoter upstream of the target genes [38,62]. Here, two NF-Y and one Sp1 binding sites were identified within a 205 bp region on a human SQLE promoter [38]. In mice, the SQLE gene is 20.5 kilobase pairs in length and regulated by SREBP2, NF-Y, and YY1; the two putative NF-Y sites present in the SQLE promoter that are conserved in the human and rat SQLE promoters [58]. Moreover, the activity of SREBP is also controlled by a wide range of stimuli such as lysophosphatidylcholine, betulin, hypoxia, tumor suppressor p53, the activation of Akt, MAPK pathways, and the inhibition of AMPK signaling [9,49].

### 4.3. Post-Transcriptional Regulation

In addition to regulation at a transcriptional level, the rapid alteration of cholesterol synthesis requires posttranscriptional control: SQLE is directly regulated by its end product cholesterol via ubiquitination-mediated degradation [10,20,52]. The ubiquitin–proteasome system is an important member in the post-translational regulation system and has been recognized as an instrumental regulator in cholesterol homeostasis [63]. The post-translational regulation of SQLE by its end product was later evidenced to be mediated by the E3 ubiquitin ligase MARCH6 (Figure 3) [35,64]. MARCH6 (also known as TEB4 or RNF176) is an evolutionarily conserved polytopic protein that resides in the endoplasmic reticulum [65]. It consists of 910 amino acids and weighs 103 kDa. MARCH6 contains 14 transmembrane domains and eight cytosolic regions with two functional domains, an N-terminal catalytic RING domain and a C-terminal regulatory element, which both face the cytosol [65,66,67,68]. It is established that the N-terminal RING domain of MARCH6 acts in conjunction with the E2 enzyme UBC7 and specifically catalyzes k48-specific ubiquitin–ubiquitin linkage, while the C-terminal element is required for its auto-ubiquitination [67,69]. Interestingly, MARCH6 is an ER degradation substrate itself and can promote its own degradation in a RING finger- and proteasome-dependent manner [69]. Through the inhibition of its auto-ubiquitination, cholesterol can inhibit the degradation of MARCH6 [70]. It is known that MARCH6 is an E3 ligase that participates in ER-associated degradation (ERAD) [71] and can thereby promote the ERAD of SQLE [72].

ERAD provides the major mechanism for protein quality control at the ER membrane, facilitating the dislocation of proteins from the ER for degradation by the proteasome within the cytosol [73]. The ERAD process involves the recognition of substrates in the lumen and membrane of the ER and their translocation into the cytosol, ubiquitination, and delivery to the proteasome for degradation [74]. After determining that cholesterol could cause SQLE to degrade post-transcriptionally through MARCH6, researchers proceeded on and revealed that the regulation required the N100 of SQLE and the degradation was controlled in a RING-dependent manner [20,34,40]. Since the ERAD function is similar to other well-characterized ubiquitination reactions and is dependent on a series of reactions catalyzed by an enzymatic cascade consisting of E1-activating, E2-conjugating, and E3-ligating enzymes that recognizes and coordinates substrate position for ubiquitin modification [73,75], researchers aimed to identify specific E2 enzymes for MARCH6 in this sterol-dependent degradation machinery. Using a CRISPR/Cas9-based approach, UBE2J2 was identified as the primary ERAD-associated E2 enzyme that is essential for the MARCH6-dependent degradation of SQLE, and disturbance of cholesterol-accelerated SQLE degradation was observed when ablating UBE2J2 in multiple human cancer cell types [41]. In addition, valosin-containing protein is involved in regulating the cholesterol-accelerated degradation of SQLE, and the amphipathic helix of SQLE N100 is critical for its regulation by valosin-containing protein [42].

Further, the ubiquitination site required for cholesterol regulation of SQLE N100 was investigated. During the past years, most studies on ubiquitination have focused on the conjugation of ubiquitin to lysine residues in substrates; however, ubiquitination can also occur on cysteine, serine, and threonine residues, as well as on the N-terminal amino group of proteins [76,77]. Initially, attempts at identifying a ubiquitination site for SQLE using site-directed mutagenesis were unsuccessful. The cholesterol-regulated turnover of SQLE was retained even when all five lysine residues were substituted by arginine [20]. Later, researchers hypothesized that SQLE N100 undergoes non-canonical ubiquitination. To test this, they mutated clusters of cysteines, serines, and threonines to alanines and found that serine residues in the second half of N100 were necessary for cholesterol-accelerated degradation and that losing residues Ser-59 and Ser-61 resulted in the greatest loss of cholesterol regulation [43]. Through intricate experiments, the mechanisms were finally revealed as the following: lysine residues are deemed dispensable for SQLE degradation while the loss of serine residues impedes it; four serines (Ser59, Ser61, Ser83, and Ser87) are critical for cholesterol-accelerated degradation, with Ser-83 being a ubiquitination site; and MARCH6 is the E3 ligase responsible for it while UBE2J2 is the likely E2 ubiquitin-conjugating enzyme mediating this process [43].

## 5. Links with Ferroptosis

Ferroptosis is a non-apoptotic mechanism of cell death that is driven by overwhelming membrane lipid peroxidation and is characterized by the requirement for the redox-active metal iron [78,79]. There is an intricate balance between ferroptosis execution and ferroptosis defence systems in cells (Table 2), and the result of ferroptosis-promoting cellular activities significantly overriding the antioxidant-buffering capabilities provided by ferroptosis defence systems is the occurrence of ferroptosis [78]. An important driver of ferroptosis are the peroxides formed on polyunsaturated fatty acids (PUFAs), while the uptake and metabolism of PUFAs and the synthesis of PUFA phospholipids crucially shape cellular sensitivity to ferroptosis (Table 2) [80]. As a vital lipid, cholesterol has been repeatedly associated with ferroptosis [81]. The intermediate metabolites of the mevalonate pathway play an important role in ferroptosis [81]. For example, IPP is required for the synthesis of selenoproteins, including GPX4, the most important antiferroptotic protein; FPP is a precursor of ubiquinone or coenzyme Q, a strong inhibitor of ferroptosis. In addition, the master lipogenesis regulator SREBP2 can directly induce transcription of the iron carrier transferrin, reducing intracellular iron pools, reactive oxygen species, and lipid peroxidation, thereby conferring resistance to inducers of ferroptosis [82]. Also, resistance to ferroptosis is a feature of metastatic cells and cholesterol can enhance the resistance of metastatic cells to ferroptosis [83].

Particularly, the interaction between SQLE and ferroptosis has been reported in recent years. SQLE can be stabilized by unsaturated fatty acids and this appears to occur through the reduction in ubiquitination by MARCH6 as well [35]. In one study, the stabilization of SQLE by fatty acids appeared to be mediated solely by unsaturated fatty acids, including PUFAs [40]. Therefore, in anaplastic large cell lymphoma cells that do not express SQLE and depend on exogenous cholesterol for their growth, squalene accumulates and alters the cellular lipid profile with antioxidant-like properties to protect these cancer cells from ferroptotic cell death [13]. More recently, the E3 ligase MARCH6 which is responsible for SQLE degradation was shown to suppress ferroptosis [14,66]. This effect was mediated through recognition of NADPH by its C-terminal element and the subsequent upregulation of its catalytic activity [14]. Surprisingly, ferroptosis induction can stabilize SQLE at a post-transcriptional level, while an iron chelator reduces SQLE levels in wild-type cancer cells [14]. Perhaps because of these established studies, SQLE has been categorized as falling into “ferroptosis regulators” or “ferroptosis genes” in some research [84,85,86]. These studies strongly suggest an innate mechanistic link between SQLE and ferroptosis, which deserves further investigation.

## 6. The Links between SQLE and Cancer

Cancers are complex diseases that have ranked in second place in the causes of deaths globally and are characterized by their acquired capabilities for sustaining proliferative signaling, evading growth suppressors, resisting cell death, and enabling replicative immortality [87,88]. The connection between dysregulated metabolism and tumorigenesis is a hot topic, and changes in specific metabolites have been revealed to cause genetic and protein expression alterations in cancer cells and vicinal non-transformed cells to facilitate biomass production, proliferation, and tumor expansion [89]. Reprogrammed lipid metabolism plays an important role in providing energy, macromolecules for membrane synthesis, and lipid-mediated signaling during cancer progression [90]. It has been revealed that tumor cells require excess cholesterol and intermediates of the cholesterol biosynthesis pathway to maintain cell proliferation, which is possibly the cause for the substantial cholesterol requirement for membrane synthesis [15]. Meanwhile, the effects of elevated cholesterol levels are not always detrimental [25,37]. For example, high serum levels of cholesterol have been reported to result in cholesterol accumulation in natural killer cells and increase the anti-tumor functions of them; activated effector functions of these cells were observed and reduced growth of liver tumors in mice were recorded [91]. Therefore, there is a complex linkage between cholesterol metabolism and cancer, and abnormalities in cholesterol biosynthesis have been strongly associated with tumorigenesis [15,92].

**Figure 4:**
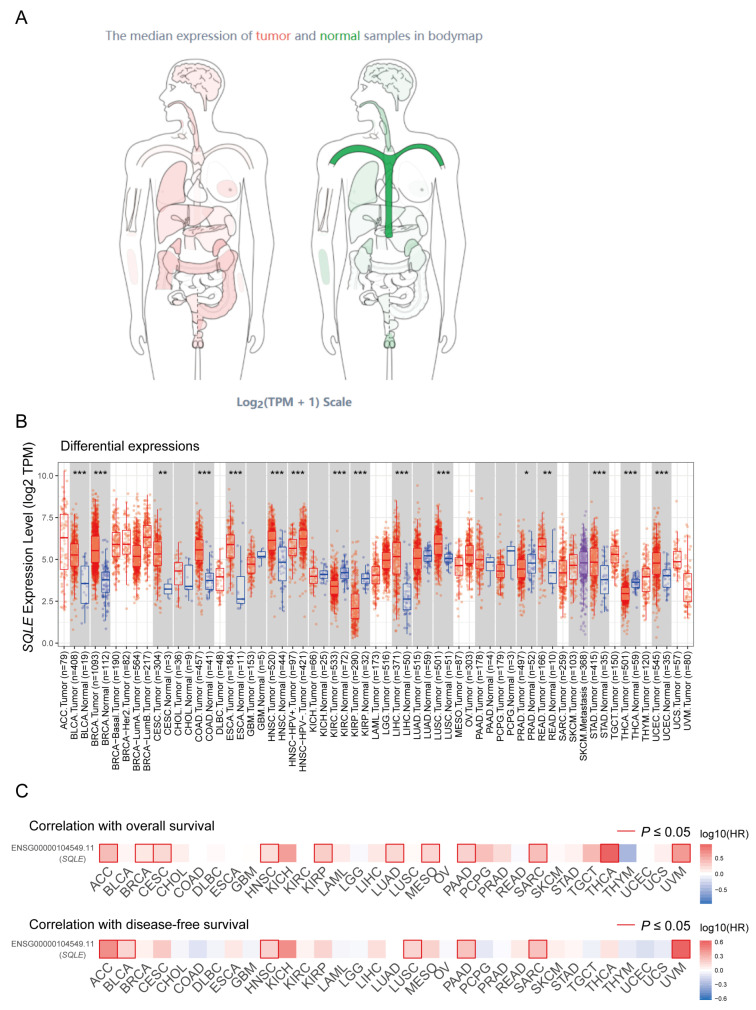
**The expression and prognostic correlations of SQLE in cancer patients**. (**A**) An overview of the differential expression of SQLE in human cancers. (**B**) The differential expression of SQLE in 33 types of human cancers. (**C**) Correlations between SQLE and overall survival and disease-free survival in cancer patients. Analyses are based on data from TCGA datasets. * *p* < 0.05, ** *p* < 0.01, *** *p* < 0.001.

In general, cholesterol is beneficial for cancer growth and development, and increased endogenous cholesterol synthesis and high cholesterol exposure both favor cancer progression [92]. As the second rate-limiting enzyme in cholesterol biosynthesis, SQLE is a fascinating enzyme that plays a vital role in human cancers [21]. After the identification of the crystal structure of SQLE in 2019, cancer research on SQLE began to boom (Table 3). The widespread functions of SQLE in cancers may be largely attributed to the hypoxic microenvironment of solid tumors due to their poorly vascularized cores [93] and the special phenomenon that SQLE undergoes of partial degradation under hypoxic conditions and transformation into a truncated form that is constitutively active [36]. In fact, SQLE has been demonstrated to be upregulated in a variety of cancer types (Figure 4A,B) and there are significant correlations between SQLE and prognoses of cancer patients (Figure 4C). In a pan-cancer analysis, the aberration of genes in the sterol synthesis pathway was prevalent and most of these genes were found altered in 4746 queried patients; the mutation rate of SQLE was the highest among all, and the amplification rate was as high as 6% in the queried cases across all cancer types [15]. In metastatic lesions, the amplification rate of SQLE can be as high as 24%, and this amplification is associated with enhanced expression [47]. This fact implies that SQLE is a bona fide oncogene in cancers and should be studied in great detail for the purpose of identifying potential druggable targets in specific malignant tumors [94]. The effects of SQLE on different tumors are summarized in Table 3 and discussed as follows.

### 6.1. Colorectal Cancer

The association between serum levels of cholesterol and colorectal cancer (CRC) is controversial. Some studies suggested that low cholesterol levels were associated with an increased cancer risk [125], while others concluded that high serum cholesterol levels could increase the risk of CRC [126]. In the CRC cohort from The Cancer Genome Atlas (TCGA), patients with a high expression of cholesterol metabolism genes had worse tumor-free survival than those with a low one, and lowering cholesterol using atorvastatin could enhance the anti-tumor effect of 5-FU on colorectal cancer cells [127]. As a rate-limiting enzyme in cholesterol biosynthesis, SQLE has been studied in CRC and the studies have shown conflicting results [11,12,109,113]. Compared to normal tissues, SQLE was downregulated in tumor tissues in the study by Jun S et al. [113] and upregulated in tumor tissues in others [11,12]. To clarify the clinicopathologic implications of SQLE in colorectal cancer patients, Kim J et al. collected samples from an independent cohort involving 143 CRC patients and performed immunohistochemistry [109]. The results showed that patients with high SQLE expression had shorter recurrence-free survival and poorer overall survival [109]. To be more specific, positive SQLE expression at the invasive front was correlated with a significantly greater presence of lymphovascular invasion, deeper invasion depth, more frequent regional lymph node metastasis, and more advanced tumor staging than in patients with low SQLE expression levels [109]. In CRC, SQLE-related control of cholesterol biosynthesis was highly upregulated in CRC patients and associated with poor prognosis [11]. Mechanistically, SQLE could promote CRC proliferation through the accumulation of calcitriol and stimulation of CYP24A1-mediated MAPK signaling [11]. However, the study by Jun SY et al. led to a different conclusion [113]. They concluded that SQLE reduction aggravates CRC progression via the activation of the β-catenin oncogenic pathway [113]. Glycogen synthase kinase-3β (GSK-3β) is an evolutionarily conserved serine/threonine kinase that is involved in multiple signaling pathways and is implicated in different diseases including inflammation, neurodegenerative disease, diabetes, and cancers [128]. The tumor suppressor protein p53 acts as a sequence-specific transcription factor and is capable of binding to defined DNA sequences within the genome, being a barrier against cancer initiation and progression [129]. Cholesterol- or siRNA-resulted SQLE reduction could dissociate the interaction of GSK-3β and p53, leading to GSK-3β inhibition and p53 degradation, thereby accelerating the malignant conversion and invasiveness of tumor cells by the induction of the epithelial–mesenchymal transition [113]. Consistently, in the study by Li C et al., colon-specific SQLE transgenic mice showed increased tumorigenesis, enriched pathogenic bacteria, and impaired gut barrier function [12]. In their study, SQLE suppressed apoptosis and promoted cell cycle progression and de novo cholesterol biosynthesis in CRC cell lines [12]. The blockade of SQLE using terbinafine can also decrease the fecal fungal load and numbers of operational taxonomic units and suppress myeloid-derived suppressor cell expansion [119]. Meanwhile, terbinafine can directly suppress CRC cell growth by disrupting the pentose phosphate pathway to interrupt nucleotide biosynthesis [119]. These conflicting results may be due to the fact that SQLE has disparate effects on CRC growth and metastasis or the different animal models used in the studies. Anyway, further conclusions remain to be drawn in future research.

### 6.2. Hepatocellular Carcinoma

Hepatocellular carcinoma (HCC) is the sixth most common cancer and the third leading cause of cancer-related deaths worldwide [130]. Non-alcoholic fatty liver disease (NAFLD) is a risk factor for the development of HCC [131]. NAFLD can progress to non-alcoholic steatohepatitis (NASH) in 20–30% of cases, and approximately 20–25% of NASH cases progress to cirrhosis, which is the leading risk factor for HCC development [131,132,133,134]. During the past years, patients with NAFLD have continued to increase with the epidemics of obesity, with an estimate of one-third of the global adult population developing NAFLD, and NAFLD is estimated to become the most prevalent etiology of HCC [135,136]. It is reported that the addition of high cholesterol in high-fat diets can cause NASH in mice and augment carcinogenesis by increasing the multiplicity and size of tumors [106]. When exploring the possible etiology, SQLE was identified to be an upregulated gene in NASH and steatosis HCCs when compared to an adjacent non-tumorous liver [106]. In other research, SQLE was also an elevated gene and protein in NAFLD-associated HCC/HCC tissues compared to adjacent normal tissues [105,123]. The oncogenic role of SQLE in NAFLD-HCC cell lines was evaluated using hepatocyte-specific *Sqle* transgenic mice [105]. SQLE was evidenced to promote cell growth, regulate cell cycle progression, and inhibit apoptosis in NAFLD-HCC cell lines, and these effects were induced via intracellular cholesterol/cholesteryl ester accumulation [105]. PI3K/AKT/mTOR signaling is a crucial intracellular pathway in regulating fundamental cellular functions, including cell growth, motility, survival, metabolism, and angiogenesis [137]. Hyperactivation of the PI3K/AKT/mTOR pathway occurs in nearly all malignant tumors and PTEN is one of the most frequent upstream alternative sites [137]. In one study, the authors verified that SQLE exerted its oncogenic effects on NAFLD-HCC cells through the activation of the PTEN/PI3K/AKT/mTOR pathway [105]. Inhibition of SQLE also suppresses the proliferation of HCC cells by inhibiting mTORC1 signaling via the activation of AMPK, and inhibition-induced mTORC1 signaling can be potentiated by sorafenib, a first-line targeted drug for HCC [111]. Transforming growth factor-β (TGFβ) is an important secretory cytokine and the signaling of the TGFβ pathway is involved in the regulation of cell proliferation, differentiation, invasion, migration, and apoptosis; the transduction of TGFβ signaling occurs via the suppressor of mothers against decapentaplegic (SMAD) and non-SMAD pathways that are mediated by different ligands [138]. SQLE expression is indispensable for HCC cell growth, migration, and F-actin assembly, while the pro-tumorigenic effect of SQLE on HCC is dependent on the activation of TGF-β/SMAD signaling [123].

### 6.3. Breast Cancer

Breast cancer (BRCA) has surpassed lung cancer to become the number one in terms of cancer incidence and cancer-related mortality for women in the world [130]. SQLE is upregulated in BRCA and is significantly inversely correlated with distant metastasis-free survival in stage I/II BRCA cases [96,110]. Moreover, SQLE is an indicator of unfavorable overall survival in BRCA patients and a high expression of SQLE is associated with a high tumor grade and Her2+ status [94,110]. Meanwhile, SQLE gene expression shows a high correlation with its locus copy number in BRCA, making it a bona fide oncogene [94]. A short open reading frame constitutes ≤300 bases and encodes a microprotein or short open reading frame-encoded protein which comprises ≤100 amino acids [139]. In the research by Polycarpou-Schwarz M et al., they identified the transmembrane microprotein, CASIMO1, which is localized in endosomes and modulates the proliferation, migration, and cell cycle progression of tumor cells [45]. Immunoprecipitation and mass spectrometry were performed to search for CASIMO1-interacting proteins and SQLE was identified as one of them [45]. CASIMO1 knockdown can decrease SQLE protein levels and decrease ERK phosphorylation, resulting in lipid droplet accumulation and reduction in the proliferation rate of tumor cells; the overexpression of SQLE can rescue this proliferation phenotype upon CASIMO1 knockdown [45]. Interestingly, the loss of SQLE by either siRNA or terbinafine caused a reduction in cell proliferation [45]. In breast cancer, lnc030 cooperated with poly(rC) binding protein 2 to stabilize SQLE mRNA, resulting in an increase in cholesterol synthesis, which in turn activated PI3K/Akt signaling, which is involved in the self-renewal of breast cancer stem cells to keep their stemness [46]. Tang W et al. identified a ferroptosis-related risk signature including SQLE in BRCA patients and found that the risk score was an independent prognostic factor [84]. Endocrine therapy is an established efficacious treatment for estrogen receptor-positive (ER+) breast cancers, causing a reduction in recurrence rates and increased survival rates [140]. Researchers from the Breast Cancer Now Toby Robins Research Centre revealed that cholesterol synthesis-related genes including MSMO1, EBP, LBR, and SQLE were upregulated in long-term estrogen-deprived BRCA cells; meanwhile, in silico analysis of two independent studies of primary ER+ BRCA patients treated with neoadjuvant aromatic inhibitors showed that the increased expression of these genes was significantly associated with a poor response to endocrine therapy and poor recurrence-free survival [101]. Moreover, SQLE is a race- and survival-related gene in breast cancers that is more highly expressed in African women compared to their Caucasian counterparts and indicates poor survival [100]. This conclusion was initially drawn from a small cohort and later validated using a larger cohort involving nearly 1000 cases [141]. More recently, Hong Z et al. discovered that the inhibition of SQLE can lead to the accumulation of squalene, thus inducing ER stress and activating the WIP1–ATM axis, therefore increasing the radiosensitivity of BRCA cells [114].

### 6.4. Head and Neck Squamous Cell Carcinoma

Head and neck squamous cell carcinoma (HNSCC) develops from the mucosal epithelium in the oral cavity, pharynx, and larynx, and it is the most common malignancy that arises from the head and neck [142]. SQLE was identified as a therapeutic target in cholesterol biosynthesis for HNSCC by a research team from Shanghai Ninth People’s Hospital [48]. In their research, SQLE mRNA was elevated in HNSCC cell lines compared to normal human oral epithelial cells and the accumulation of squalene, the substrate of SQLE, inhibited HNSCC cell growth [48]. Mechanistically, SQLE is upregulated by the inhibition of histone methyltransferase EZH2, and the inhibition of SQLE can largely enhance the sensitivity of HNSCC cells to EZH2 inhibitors [48]. Later, results from another team revealed that high SQLE expression in HNSCC was associated with the TNM stage, distant metastasis, and poor survival [117]. SQLE was upregulated in tumor tissues compared to normal peritumor tissues and the proliferation phenotype upon SQLE knockdown was also the same as in the previous research [112,117]. In addition, the upregulation of SQLE is associated with cisplatin resistance and the depletion of SQLE can potentiate cisplatin sensitivity in resistant HNSCC cells both in vitro and in vivo [122]. Using a luciferase reporter assay, researchers showed that TCF4 was enriched in the SQLE promoter region and that the β-catenin/TCF4 complex transcriptionally modulated SQLE expression in HNSCC cells upon cisplatin exposure [122]. Furthermore, SQLE inactivation can suppress the global c-Myc transcriptional program in HNSCC cancer stem cells, thereby reducing their stemness and tumorigenic characteristics [122].

### 6.5. Non-Small Cell Lung Cancer

Lung cancer ranks as the number one cause of cancer-related deaths according to Global Cancer Statistics, and non-small cell lung cancer (NSCLC) is its major subtype [130]. Squamous cancer (SCC) is a subtype of NSCLC and SQLE was identified as a differentially expressed gene in this cancer long ago [95,98]. The expression of SQLE mRNA and protein in lung SCC tissues was significantly higher than in pericarcinomal tissues and the expression of it was closely correlated with poor differentiation, clinical stages, and lymphatic metastasis and negatively associated with overall survival rate [98]. Later on, it was revealed that the overexpression of SQLE promoted lung SCC cell proliferation, migration, and invasion through interaction with the ERK signaling pathway [108]. Comparatively, the research on SQLE in adenocarcinoma, a major type of NSCLC, is relatively limited. In adenocarcinoma, SQLE inhibition can lead to the enhancement of radiosensitivity [114]. Most recently, researchers discovered that physical exercise could significantly inhibit cholesterol metabolism by inhibiting SQLE expression and reverse the immuno-cold tumor-immune microenvironment, which is interesting [120]. The predicted result using a public cohort from the GEO database showing that SQLE might be associated with immunotherapeutic responses in NSCLC patients and the validated result that SQLE was downregulated in cases with a poor response linked cholesterol metabolism to immunotherapy [120], which deserves further exploration.

### 6.6. Prostate Cancers

Prostate cancers rank the second in the incidence of cancers for males worldwide [130]. SQLE is overexpressed in advanced prostate tumor tissues and its expression is correlated with poor survival [47]. In prostate cancer cells, SQLE was negatively regulated by miR-205 and downregulated SQLE not only reduced cholesterol synthesis, but also reduced cholesterol intake [47]. Androgen deprivation therapy that inhibits or blocks the production or action of androgens that are male hormones has been the mainstay for treating advanced/metastatic or recurrent prostate cancer for several decades [143]. SQLE inhibition by short hairpin RNA can block cell proliferation and overcome resistance to second-generation androgen receptor inhibitors [47]. Inhibition of SQLE by FR194738 was also effective in inhibiting prostate cancer and proceeded to a castration-resistant stage in a preclinical setting [50]. The Gleason score assesses the degree of prostate cancer cell abnormality in a histological biopsy, with a lower Gleason score indicating a closer resemblance of the cancer cells to normal cells and a higher likelihood of slow growth and limited metastatic potential [143]. In another study using prostate patient samples from three well-described prospective cohort studies, researchers found that the expression of SQLE was associated with lethal cancer in the three cohorts and male patients with a high SQLE expression were 8.3 times more likely to have lethal cancer than those with a low SQLE expression [103]. They also observed that a high mRNA expression of SQLE was strongly associated with a higher Gleason grade [103]. Using the same cohorts, researchers further discovered that a higher Gleason grade was associated with a lower LDLR, lower SOAT1, and higher SQLE [104]. Since LDLR is a gene-encoding receptor responsible for cholesterol intake, SOAT1 is for esterification, and SQLE is for cholesterol biosynthesis [37], a conclusion was drawn as the following: prostate cancers that progress to lethal disease rely on de novo cholesterol synthesis via SQLE, rather than transcellular uptake via LDLR or cholesterol esterification via SOAT1 [104].

Castration-resistant prostate cancer (CRPC) is an advanced stage of prostate cancer and has a high morbidity and mortality [144]. Normally, reducing the level of circulating testosterone can effectively control the growth and spread of castration-sensitive prostate cancer cells that rely on androgens for their growth and survival [143]. However, most patients will inevitably progress to the castration-resistant stage, when prostate cancer continues to progress despite decreased levels of testosterone [143]. SQLE was upregulated in CRPC patients compared to castration-sensitive prostate cancer patients and CRPC patients with poor survival were characterized by a high level of SQLE [50]. A positive relationship was observed between the copy number amplification of SQLE and its mRNA expression in metastatic CRPC samples [50], making SQLE a potential oncogene. This was further evidenced by functional experiments showing that SQLE is necessary for CRPC tumor cell growth in vitro and in vivo [50]. An evident character of metastatic CRPC is the prevalence of PTEN/p53 loss [143,144], so PTEN–/– and TP53–/– CRPC cells were used. The high expression of SQLE in CRPC cells was caused transcriptionally by PTEN/p53 deficiency via the activation of SREBP2 [50]. Also, PTEN loss-activated PI3K/Akt/GSK3β signaling stabilized the SQLE protein throughout the N100 region [50]. Lipid rafts are cholesterol- and sphingolipid-enriched specialized membrane domains within the plasma membrane and act as recruitment platforms for signaling proteins [145]. The ablation of SQLE reduced both intracellular free cholesterol and cholesteryl ester levels and significantly decreased raft cholesterol concentrations [50]. This could induce the expression of cleaved caspase-3 and reduce the expression of PCNA, phospho-Akt, and phospho-ERK1/2, while exogenous cholesterol could restore them [50].

### 6.7. Pancreatic Cancer

Pancreatic cancer is the seventh leading cause of cancer-related deaths worldwide and is a highly fatal malignancy [146]. Most pancreatic cancers are characterized as pancreatic ductal adenocarcinoma (PDAC) and the 5-year survival rate is less than 10% [146]. SQLE was upregulated in PDAC cell lines, and an elevated level of SQLE in tumor tissue was correlated with a poor prognosis in pancreatic cancer patients [116]. Downregulation of SQLE significantly suppressed cancer cell proliferation and enhanced chemotherapeutic sensitivity [116]. In PDAC cell lines, SQLE was found to be associated with radioresistance [99]. In pancreatic adenocarcinoma, the potential oncogenic role of SQLE was established using public transcriptomic data from the TCGA database [118]. SQLE expression was upregulated in pancreatic adenocarcinoma tumor tissues compared to normal tissues, and it predicted poor DFS and OS in cancer patients; amplification was the dominant type of mutation and was closely associated with OS, DFS, and PFS [118]. SQLE was also significantly associated with tumor-immune cell infiltration, immune checkpoints including PD-1 and CTLA-4, and biomarkers of the tumor-immune microenvironment [118]. As a ferroptosis-related gene, SQLE can be incorporated into a gene signature and constitute an independent prognostic factor [86]. Most recently, a study by Xu R et al. revealed that the pro-tumoral effect of SQLE was exerted through the following mechanism: it enhanced de novo cholesterol biosynthesis and maintained lipid raft stability, thereby activating the Src/PI3K/Akt signaling pathway, and the inhibition of SQLE could lead to squalene accumulation-induced ER stress and subsequent apoptosis [121].

### 6.8. Glioblastoma

Glioma is the most prevalent type of primary tumor that derives from the central nervous system, and glioblastoma (GBM) constitutes its most malignant and lethal type [147]. In GBM, SQLE was identified to be lowly expressed in temozolomide-resistant glioma cells and involved in the ERK-mediated temozolomide resistance of glioma cells, while overexpression of SQLE significantly inhibited the migration and invasion of tumor cells [115]. Furthermore, SQLE was confirmed to have a significant correlation with tumor-infiltrating lymphocytes and immunomodulators, which highlighted that SQLE could be a potential target and biomarker for the therapy and prognosis of patients with GBM [115]. In GBM, NR4A2 activates SQLE to dysregulate cholesterol homeostasis in non-tumor stromal microglia, and the pharmacological blockade of NR4A2 or SQLE can augment immune checkpoint blockade therapeutic efficacy; particularly, terbinafine and anti-PD1 induced tumor regression and prolonged survival for GL261-luc-bearing mice compared with each monotherapy or treatment control [51].

### 6.9. Other Cancers

In addition to the cancers mentioned above, SQLE also participated in the progression of other cancers such as small cell lung cancer, glioma, esophageal cancer, and oral squamous cell carcinoma (OSCC) [44,97,115,148]. In small cell lung cancer, the viability of tumor cells can be inhibited by NB598, an inhibitor of SQLE [148]. This result is not from the reduction in cholesterol synthesis, but from the accumulation of squalene stored in lipid droplets [148]. In esophageal squamous cell carcinoma cells, SQLE was identified as the direct downstream target gene of miR-133b by luciferase gene reporter assay and its knockdown could inhibit the proliferation, invasion, and metastasis of cancer cells, while suppressing the epithelial-to-mesenchymal transition [44]. Stäubert C et al. found that the cholesterol biosynthetic pathway was upregulated in the daunorubicin-resistant leukemia cell line CEM/R2 compared to its wild-type derivate [102]. Although an increased flux was through lanosterol and not the cholesterol pool in resistant CEM/R2 cells, the application of terbinafine could suppress tumor growth [102]. In OSCC, SQLE was elevated in cancer tissues, and overexpressed SQLE led to a high cholesterol concentration, which induced the transformation of CD4+ T cells to Treg cells and promoted tumor development [124]. Also, terbinafine can decrease cell numbers in cultured human OSCCs in a concentration-dependent fashion, inhibiting DNA synthesis and inducing G0/G1 cell cycle arrest [97].

## 7. Inhibitors and Clinical Therapeutic Implications

As rate-limiting enzymes in cholesterol biosynthesis, HMGCR and SQLE have been considered druggable and focused on by researchers, with extensive attention paid to the former while the latter has been relatively less well studied [15]. As famous and developed inhibitors of HMGCR, statins mediate the reduction of cholesterol and lead to interruption of their cell membrane structure and related biological functions, such as angiogenesis, apoptosis, and autophagy. Accumulating preclinical and clinical trials of statins in different cancers suggested an overall beneficial role of statins with a favorable safety profile in cancer treatment and prevention [149]. In some established meta-analysis studies, statin use was correlated with reduced risk of cancer development and cancer-specific mortality in cancer patients and was associated with favorable survival outcomes [150,151,152]. However, due to the upstream position of HMGCR in sterol synthetic pathways, statin application can also lead to the broad inhibition of the entire pathway, decreasing not only cholesterol levels, but also additional non-sterol products of the isoprenoid pathway, such as dolichols, ubiquinone, and various isoprenylated proteins [153,154]. In fact, statin-related toxicities, such as hepatic transaminase increases and myopathies, have been widely reported [155]. More importantly, it was reported that the production of non-steroidal products in the cholesterol pathway, such as coenzyme Q, is necessary for tissue-resident T cells to potentiate mitochondrial respiration and augment anti-tumor immunity [8]. In a tumor microenvironment, tissue-resident memory CD8+ T cells deploy a range of adaptations to maintain a heightened state of activation; they are characterized by an increased activity of the transcription factor SREBP2 and become reliant on non-steroidal products of the mevalonate–cholesterol pathway, such as coenzyme Q [8]. This prompted us to wonder whether the inhibition of SQLE, another rate-limiting enzyme downstream in cholesterol biosynthesis, is able to generate survival benefits in cancer patients while overcoming these side effects.

SQLE is one of the most promising therapeutic targets for drug development in cholesterol biosynthesis [156]. Earlier research has investigated SQLE as a pharmacological target for reducing cholesterol levels [148], hence being another attractive target for selective therapy [148]. Naftifine was the first SQLE inhibitor, developed as an antifungal agent; after naftifine, other compounds were discovered, such as terbinafine, butenafine, and SDZ-SBA-586 [15]. In 1990, NB-598 was synthesized [157]. The effects of NB-598 on de novo cholesterol biosynthesis have been analyzed. The process was monitored after exposing cells to ^13^C_2_-labeled acetate. Co-treatment of labeled acetate with 1 μM NB-598 resulted in the dramatic suppression of the entire population of labeled isotopomers of cholesterol; also, a dramatic accumulation of squalene in a dose-dependent fashion was observed after the treatment of cancer cells using NB-598 [148]. Another synthetic compound that inhibits SQLE is FR194738, derived from NB-598, but it has improved lipophilic and pharmacokinetic properties [158]. FR194738 has similar potency to NB-598 and can effectively inhibit cholesterol synthesis [15].

As one of the earliest developed inhibitors, terbinafine has been tested in preclinical studies and retrospective clinical studies. The anti-tumor effect of terbinafine has been observed when treating BRCA, NSCLC, HCC, leukemia, CRC, prostate cancer, pancreatic cancer, and OSCC [12,47,97,102,111,114,121]. Other inhibitors such as NB598 and FR194738 also showed anti-tumor effects but attracted less attention from researchers [47,50,114,121,122,123]. In BRCA, terbinafine enhanced the radiosensitivity of cancer cells and promoted their sensitivity to PARP inhibitors [114]. In this study, NB598 showed a similar effect to terbinafine [114]. In HNSCC, terbinafine enhanced cisplatin sensitivity: terbinafine alone partially reduced tumor lesion area and metastatic lymph nodes, while the combination of cisplatin and terbinafine resulted in a more robust inhibitory effect [122]. In particular, terbinafine in combination with 5-FU or oxaliplatin, chemotherapy drugs commonly used in CRC, synergistically suppressed CRC growth in vitro and in vivo [12].

The anti-tumor effect of terbinafine has also been observed in cancer patients. In a case series of four, late-stage, heavily pretreated, prostate cancer patients receiving orally administered terbinafine as off-label, individual, clinical interventions, a PSA decline was observed in three of the four patients after two weeks of treatment [47]. More explicit effects of terbinafine on prostate cancer were observed in a retrospective cohort study involving patients from the Swedish Cancer Registry [107]. In that cohort, patients who received systemic treatment of terbinafine had a decreased risk of death from prostate cancer and a decreased risk of death overall [107]. In a CRC cohort from the same national institution, a reduced risk of cause-specific death was observed in patients who received systemic terbinafine compared to the controls [119]. In this cohort, systemic use of terbinafine was also associated with a lower risk of metastasis [119].

Considering the inspiring results of SQLE inhibitors, especially terbinafine, in preclinical studies and their benefits that were shown in retrospective cohort studies, it seems paradoxical that almost no prospective clinical studies on these drugs have been registered to date [159,160,161,162]. There are several possible reasons. First of all, there may be intolerable adverse events. It has been proposed that terbinafine induces fungal cell death not only due to the depletion of ergosterol but also the toxic accumulation of squalene [9]. In humans, the development of NB598 and FR194738 was also interrupted due to the accumulation of squalene in the skin upon treatment [163]. In addition, the high concentration of the inhibitors used in preclinical studies may not be readily translated to effects in clinical applications. The inhibitory activity of terbinafine for mammalian SQLE is several orders of magnitude lower than for fungal SQLE [164]. Indeed, terbinafine was found to be a far less potent inhibitor, with a much higher IC50 compared to NB-598 or Cmpd-4, which may be attributed to its chemical structure that could have led to suboptimal non-polar contacts [19,21]. Even so, the study by Nagaraja R et al. demonstrated that significant toxicities of these inhibitors arose at exposures well below the predicted levels needed for anti-tumor activity [153]. Furthermore, cancer cells can acquire necessary cholesterol from their environment even when their de novo biosynthesis pathway is blocked, so it is not always enough to inhibit only one pathway. This is evidenced by the observation that hypercholesterolemia can impair the anticancer efficacy of SQLE targeting therapy in vivo [50]. Importantly, supportive evidence for a protective effect of SQLE inhibition on cancer risk has not always been consistent. The role of SQLE in CRC has differed in different studies, for example in [12,113]. Although terbinafine as an off-label intervention has shown clinical benefits in cancer patients, there might be some non-negligible issues that have not been reported. Finally, statins may have irreplaceable advantages: the inhibition of HMGCR by statins depletes the pools of mevalonate, IPP, FPP, and geranylgeranyl pyrophosphate in cells, which can lead to reduced RAS and Rho isoprenylation, signal transduction, and DNA synthesis, which are important functional consequences of statins in the treatment of cancer [6]. Nevertheless, inspirational data could make SQLE inhibitors such as terbinafine promising adjuvants or anti-tumor drugs, if their shortcomings could be overcome successfully.

Drug repurposing is also called drug repositioning, reprofiling, or retasking and is a strategy for identifying new uses for approved or investigational drugs that are outside the scope of the original medical indication [165]. Given the high attrition rates, substantial costs, and slow paces of the discovery and development of new drugs, repurposing “old” drugs to treat both common and rare diseases is increasingly becoming an attractive proposition, as it provides an opportunity to accelerate drug development and is characterized by a lower risk of failure, a reduced time frame for drug development, less investment, and the possibility of revealing new targets and pathways that can be further exploited [165,166]. A famous example for drug repurposing is metformin, the most commonly used glucose-lowering agent during the past 60 years, which has been reported to promote anticancer immunity through the modulation of the tumor immune microenvironment [167]. Enlightened by this, we can expect an increase in the investigation of SQLE inhibitors such as terbinafine in clinical studies in the coming years. Given the androgen-dependent nature of prostate cancer and the fact that cholesterol can be converted into androgen in cancer cells [143,168], we expect a breakthrough to be made in researching and treating prostate cancers. Alternatively, considering the important role intestinal microbiota plays in CRC development and progression [12,119], and the site-specific effect of antibiotics on colorectal carcinogenesis [169], a breakthrough may also be obtained in CRC.

## 8. Conclusions

SQLE is a rate-limiting enzyme in the cholesterol biosynthesis pathway and has not received considerable attention up to now. It catalyzes the first oxygenation step, the conversion of squalene to 2,3-epoxysqualene, in cholesterol biosynthesis and its expression is strictly controlled by regulators of different layers. Important regulators of SQLE expressions including cholesterol, SREBPs, and MARCH6. Overexpression of SQLE in tumor tissues and correlations between SQLE and patients’ prognoses have been observed in many cancers. Most of the time, SQLE plays a pro-tumoral role in various cancers through plenty of signaling pathways. According to the literature, the inhibition of SQLE by siRNA or compounds can both retard the development of tumors. Although preclinical studies and retrospective cohort studies have shown the promise of SQLE inhibitors in cancer therapy, no prospective clinical studies have been registered yet. Future efforts may be made in the translation of the research into clinical application, and it is hoped that the first breakthroughs will be made in prostate cancer and CRC.

## Figures and Tables

**Table 1:** Summary of the regulation of SQLE.

Regulation Level	Involved Molecule	Mechanism	References
End product	Cholesterol	Cholesterol-accelerated degradation is dependent on the SQLE N100 regulatory domain of SQLE and happens at the post-transcriptional level.	[18,20]
End product	Cholesterol	The interaction between cholesterol and SQLE was confirmed using a chemoproteomic strategy.	[29]
End product	Cholesterol enantiomer	Ent-cholesterol also accelerates the proteasomal degradation of SQLE via suppression of the activation of SREBP2.	[33]
Substrate	Squalene	Squalene directly binds to the N100 region, thereby reducing interaction with and ubiquitination by MARCH6.	[34]
Other	Unsaturated fatty acids	SQLE is stabilized by unsaturated fatty acids.	[35]
Other	–	Hypoxia-induced squalene accumulation promotes partial degradation of SQLE through MARCH6 to a constitutively active truncated form.	[36]
Transcription	SREBP2	When cholesterol levels are low, SREBP2 enters the nucleus to bind to the SRE sequence in the promoters of target genes and induce the expression of them.	[1,3,37]
Transcription	NF-Y	NF-Y sites were identified on an SQLE promoter and act as cofactors for transcription.	[16,38]
Transcription	Sp1	Sp1 sites were identified on an SQLE promoter and act as cofactors for transcription.	[38,39]
Transcription	YY1	A putative binding site for YY1 was predicted on an SQLE promoter.	[16]
Post-transcription	MARCH6	MARCH6 acts as an E3 ligase, and its overexpression reduces SQLE abundance in a RING-dependent manner.	[40]
Post-transcription	UBE2J2	UBE2J2 was identified as the primary E2 ubiquitin-conjugating enzyme essential for MARCH6-dependent degradation of SQLE in mammalian cells.	[41]
Post-transcription	VCP	VCP regulates SQLE N100 in a MARCH6-dependent manner.	[42]
Post-transcription	Serine residues for ubiquitination	Four serines (Ser-59, Ser-61, Ser-83, Ser-87) are critical for cholesterol-accelerated degradation, with Ser-83 used as a ubiquitination site.	[43]
Transcription	MiR-133b	SQLE is a direct target of miR-133b in esophageal cancer.	[44]
Post-transcription	CASIMO1	CASIMO1 interacts with SQLE and modulates lipid droplet accumulation in breast cancer.	[45]
Transcription	Lnc030, PCBP2	Lnc030 cooperates with PCBP2 to stabilize SQLE mRNA in breast cancer.	[46]
Transcription	MiR-205	MiR-205 controls SQLE expression through the 3′-UTR of SQLE mRNA in prostate cancer.	[47]
Transcription	EZH2	EZH2 inhibitor promotes SQLE expression by reducing H3K27me3 modification.	[48]
Transcription	P53	P53 directly represses the expression of SQLE in an SREBP2-independent manner.	[49]
Transcription	PTEN/p53	PTEN/p53 deficiency enhances SQLE expression via the activation of SREBP2.	[50]
Transcription	NR4A2	NR4A2 binds to the promoter region of SQLE to activate it in microglia.	[51]

**Table 2:** A brief summary of ferroptosis-driving and ferroptosis defence mechanisms.

Agent Types	Agent Name	Description
Ferroptosis prerequisites	PUFA-PLs	ACSL4, LPCAT3, and ACC are enzymes responsible for the synthesis and peroxidation of PUFA-PLs.
	Iron	Iron is involved in the Fenton reaction for the direct peroxidation of PUFA-PLs; it also acts as a cofactor for enzymes that participate in lipid peroxidation (such as ALOX and POR).
	Mitochondrial metabolism	Mitochondrial ROS are critical for lipid peroxidation and ferroptosis onset; electron transport and proton pumping in mitochondria are important for ATP production; and the role of mitochondria in biosynthetic pathways in cellular metabolism contributes to ferroptosis.
Ferroptosis defence mechanisms	SLC7A11–GSH–GPX4 system	This is the major cellular defence system for ferroptosis.
	FSP1–CoQH_2_ system	FSP1 is capable of reducing CoQ to CoQH2.
	DHODH–CoQH_2_ system	DHODH can reduce CoQ to CoQH2.
	GCH1–BH_4_ system	GCH1 suppresses ferroptosis through the generation of BH4 as a radical-trapping antioxidant and mediates the production of CoQH2 and PLs containing two PUFA tails.

A summary from recently published reviews by Lei G et al. [78] and Stockwell BR [79]. Abbreviations: PUFA, polyunsaturated fatty acid; PL, phospholipid; ROS, reactive oxygen species; SLC7A11, solute carrier family 7 member 11; GSH, glutathione; GPX4, glutathione peroxidase 4; FSP1, ferroptosis suppressor protein 1; CoQ, coenzyme Q/ubiquinone; CoQH_2_, ubiquinol; DHODH, dihydroorotate dehydrogenase; GCH1, GTP cyclohydrolase 1; BH4, tetrahydrobiopterin.

**Table 3:** Summary of the studies on SQLE in cancers.

Year	Tumor Type	Study Type	Prognostic Significance	Involved Pathways/Mechanisms	References
2007	NSCLC	2	–	SQLE mRNA was higher in tumor samples compared to normal lung samples.	[95]
2008	BRCA	2	DFS in stage I/II breast cancer cases was significantly inversely related to SQLE mRNA.	–	[96]
2012	OSCC	1	–	Terbinafine decreased cell viability, inhibited DNA synthesis, and induced G0/G1 cell cycle arrest.	[97]
2014	NSCLC	2	SQLE mRNA levels were negatively associated with OS.	SQLE mRNA and protein in LUSC were significantly elevated.	[98]
2014	PC	1, 2	–	11 genes including SQLE were consistently associated with radioresistance in the studied cell lines.	[99]
2015	BRCA	2	High SQLE was associated with higher mortality among luminal A BRCA.	SQLE mRNA was differentially expressed by race among luminal A breast cancers and associated with survival.	[100]
2016	BRCA	1, 2	SQLE overexpression was associated with worse OS.	SQLE was identified as a bona fide metabolic oncogene by amplification.	[94]
2016	BRCA	1	–	In primary ER+ BRCA, increased expression of SQLE were significantly associated with a poor response to endocrine therapy.	[101]
2016	LK	1	–	The cholesterol biosynthetic pathway was upregulated in daunorubicin-resistant leukemia cells.	[102]
2016	PrC	3	–	Men with high SQLE expression were more likely to have lethal cancer and tumor angiogenesis.	[103]
2017	EC	1, 2	–	SQLE is a downstream target gene of miR-133b and induces EMT.	[44]
2017	PrC	2, 3	–	PrC patients that progressed to lethal disease relied on de novo cholesterol synthesis via SQLE.	[104]
2018 April	HCC	1, 2	High SQLE expression was an independent prognostic factor associated with poor DFS.	SQLE silenced PTEN via induction of the ROS–DNMT3A axis and activated the PTEN/PI3K/AKT/mTOR pathway.	[105]
2018 August	BRCA	1, 2	–	Knockdown of CASIMO1 decreased SQLE protein, lipid droplets, and ERK phosphorylation.	[45]
2018 October	HCC	1, 2	–	SQLE was upregulated in both NASH and steatosis HCCs.	[106]
2019 April	PrC	2	–	Terbinafine decreased the risk of death from PrC and risk of death overall.	[107]
2019 March	NSCLC	1, 2	Higher SQLE indicated shorter OS in LUSC.	SQLE could interact with ERK to enhance its phosphorylation.	[108]
2019 May	CRC	2	SQLE-positive patients had shorter RFS and poorer OS than SQLE-negative ones.	–	[109]
2020 November	BRCA	1, 2	–	Lnc030 cooperates with PCBP2 to stabilize SQLE mRNA and activates PI3K/Akt signaling to govern breast CSC stemness.	[46]
2021 April	BRCA	2	High SQLE expression was associated with poor DFS and OS.	–	[110]
2021 August	CRC	1, 2	CRC patients with higher SQLE expression had shorter OS.	Inhibition of SQLE reduced calcitriol and CYP24A1, increased intracellular Ca^2+^, and suppressed MAPK signaling.	[11]
2021 August	HCC	1	–	Terbinafine and sorafenib inhibit mTORC1 signaling via AMPK activation and induce double-stranded DNA breaks.	[111]
2021 August	PC	1, 2	–	Blocking PTGS2 and SQLE suppressed the protein expression of cyclin D1 and N-cadherin and facilitated E-cadherin.	[86]
2021 August	PrC	1, 2	High SQLE is significantly associated with shorter biochemical RFS and worse RFS and OS.	SQLE expression is controlled by micro-RNA 205.	[47]
2021 June	HNSCC	1, 2	High SQLE expression was significantly associated with poor OS and PFS.	High SQLE expression promoted cell proliferation and was associated with the T stage in HNSCC patients.	[112]
2021 March	CRC	1, 2	The median survival of CRC patients with high SQLE mRNA levels was 80% higher than those with low SQLE levels.	SQLE reduction inhibited GSK-3β and p53 degradation, inducing EMT to aggravate CRC progression.	[113]
2021 May	HNSCC	1, 2	HCC patients with higher SQLE expression had poorer OS, RFS, PFS, and disease-specific survival.	Inhibition of the histone methyltransferase EZH2 strongly induced the expression of the SQLE gene.	[48]
2021 October	HCC	1, 2	–	P53 directly represses the expression of SQLE in an SREBP2-independent manner.	[49]
2022 April	BRCA	1, 2	Patients with high SQLE had worse OS and DFS.	SQLE inhibition resulted in squalene accumulation and triggered ER stress, which activated the WIP1–ATM axis.	[114]
2022 April	NSCLC	1, 2	SQLE was identified as a predictor of poor OS.	Inhibition of SQLE led to the accumulation of squalene, inducing ER stress and activating the WIP1–ATM axis.	[114]
2022 December	GBM	1, 2	Low SQLE expression was significantly associated with poor OS.	SQLE suppressed ERK-mediated TMZ chemoresistance and metastasis of GBM cells.	[115]
2022 July	PC	1, 2	PDAC patients with higher SQLE expression had shorter OS.	SQLE silencing blocked the cell cycle in the S phase.	[116]
2022 May	HNSCC	1, 2	The OS and PFS of patients with high SQLE expression were notably shorter.	SQLE overexpression mediated HNSCC progression through PI3K/Akt signaling.	[117]
2022 May	PC	1, 2	SQLE predicted poor DFS and OS.	SQLE was significantly associated with tumor immune cell infiltration and immune checkpoint expression.	[118]
2022 November	CRC	1, 2	High SQLE mRNA levels were associated with poor OS in patients with CRC.	SQLE induced cell cycle progression, gut dysbiosis, and increased secondary bile acids and suppressed apoptosis.	[12]
2022 October	CRC	1	–	Terbinafine led to nucleotide synthesis disruption, deoxyribonucleotide starvation, and cell cycle arrest.	[119]
2022 September	PrC	1, 2	SQLE expression levels were positively correlated with worse OS in patients with CRPC.	PTEN/p53 deficiency transcriptionally upregulated SQLE via activation of SREBP2 and inhibited the PI3K/Akt/GSK3β pathway.	[50]
2023 April	GBM	1, 2	–	NR4A2 activated SQLE to dysregulate cholesterol homeostasis in microglia.	[51]
2023 August	NSCLC	1, 2	–	Physical exercise could significantly inhibit SQLE expression and reverse the immuno-cold TIME.	[120]
2023 August	PC	1,2	Patients with higher SQLE expression levels had worse OS and DFS.	SQLE inhibition led to ER stress and apoptosis; SQLE activated the Src/PI3K/Akt signaling pathway.	[121]
2023 July	HNSCC	1, 2	High SQLE expression was correlated with shorter OS/DFS time.	SQLE inactivation suppressed the global c-Myc transcriptional program in CSCs.	[122]
2023 June	HCC	1, 2	–	SQLE promoted tumor growth via TGF-β/SMAD signaling, which is critically dependent on STRAP.	[123]
2023 September	OSCC	1, 2	Higher levels of SQLE expression were associated with shorter OS.	SQLE induced the transformation of CD4+ T cells to Treg cells and promoted tumor development.	[124]

Study type: 1 for preclinical, 2 for clinical retrospective, 3 for clinical prospective. Abbreviations: NSCLC, non-small cell lung cancer; BRCA, breast cancer; DFS, disease-free survival; OSCC, oral squamous cell carcinoma; OS, overall survival; LUSC, lung squamous cell carcinoma; PC, pancreatic cancer; LK, leukemia; PrC, prostate cancer; EC, esophageal cancer; EMT, epithelial–mesenchymal transition; HCC, hepatocellular carcinoma; NASH, non-alcoholic steatohepatitis; RFS, recurrence-free survival; CSC, cancer stem cell; CRC, colorectal cancer; HNSCC, head and neck squamous cell carcinoma; PFS, progression-free survival; ER, endoplasmic reticulum; GBM, glioblastoma; PDAC, pancreatic ductal adenocarcinoma; CRPC, castration-resistant prostate cancer.

## Data Availability

Not applicable.
